# Up‐regulation of miR‐497 confers resistance to temozolomide in human glioma cells by targeting mTOR/Bcl‐2

**DOI:** 10.1002/cam4.987

**Published:** 2017-01-08

**Authors:** Danhua Zhu, Ming Tu, Bo Zeng, Lin Cai, Weiming Zheng, Zhipeng Su, Zhengquan Yu

**Affiliations:** ^1^ Department of NeurosurgeryThe First Affiliated Hospital of Soochow University188 Shizi StreetCanglang DistrictSuzhouJiangsu215000China; ^2^ Department of NeurosurgeryThe First Affiliated Hospital of Wenzhou Medical UniversityWenzhouZhejiang325000China

**Keywords:** IGF1R/IRS1 pathway, microRNA, proliferation, tumor biology

## Abstract

The occurrence of an inherent or acquired resistance to temozolomide (TMZ) is a major burden for patients suffering from glioma. Recently, studies have demonstrated that microRNAs play an important role in the regulation of tumor properties in cancers. However, whether miR‐497 contributes to glioma resistance to chemotherapy is not fully understood. In this study, we showed that the expression of miR‐497 was markedly up‐regulated in TMZ‐resistant glioma cells; high miR‐497 expression level was associated with TMZ‐resistant phenotype of glioma cells. The down‐regulation of miR‐497 in glioma cells enhanced the apoptosis‐induction and growth inhibition effects of TMZ both in vitro and in vivo, whereas promotion of miR‐497 increased the chemosensitization of glioma cells to TMZ. The increased level of miR‐497 in TMZ‐resistant glioma cells was concurrent with the up‐regulation of insulin‐like growth factor 1 receptor (IGF1R)/insulin receptor substrate 1 (IRS1) pathway‐related proteins, that is, IGF1R, IRS1, mammalian target of rapamycin (mTOR), and Bcl‐2. In addition, the knockdown of mTOR and Bcl‐2 reduced the tolerance of glioma cells to TMZ. Our results demonstrated that overexpression of miR‐497 is significantly correlated with TMZ resistance in glioma cells by regulating the IGF1R/IRS1 pathway. Therefore, miR‐497 may be used as a new target for treatment of chemotherapy‐resistant glioma.

## Introduction

Human gliomas are the most frequent and most aggressive primary brain tumor that exhibits malignant aggressiveness and poor prognosis 1, 2. Currently, although surgical operation combined with radiotherapy and chemotherapy are effective remedies for the treatment of primary glioma, the prognosis of glioma patients still remains poor, with a median survival time of about 12 months 3, 4. Temozolomide (TMZ) is a second generation of alkylating agent that induces apoptosis via methylation of guanine residues 5. TMZ concurrently and after radiotherapy significantly improves the overall survival compared with radiation therapy alone and has the advantage of wide applicability and minimal additional toxicity 6, 7. It has become the current standard chemotherapeutic drug in the treatment for glioma. However, the development of inherent or acquired TMZ resistance limits its clinical application, and the mechanisms of TMZ resistance are not fully understood 8, 9. Thus, identification of new molecular targets is urgently required to fight against TMZ resistance in glioma.

MicroRNAs (miRNAs) are a class of endogenous and small noncoding RNAs with 21–23 ribonucleotides, which can induce mRNA degradation or act as repressors of translation through directly binding to a target site in the 3′‐untranslated regions of their target genes. Existing studies found that miRNA mediated gene regulation involved in multiple pathological processes including cell proliferation, differentiation, migration, survival, and tumorigenesis 10, 11. Recent evidence has shown that miRNAs also plays a vital role in chemotherapeutic resistance. For instance, miR‐873 was found to improve the chemosensitivity of glioblastoma cells to cisplatin by targeting Bcl‐2 12. MiR‐16 was reported to mediate TMZ resistance in glioma cells by modulation of apoptosis 13, highlighting miRNAs as potent chemo‐resistant modulators in the treatment of glioma.

MiR‐497 is found as a tumor suppressive miRNA in most human cancer. Such function is exerted partly by its anti‐proliferative and anti‐growth potential 14, 15, 16, 17. Previous study showed that miR‐497 induced breast cancer cell apoptosis by negatively regulating Bcl‐2 protein expression 18. Recent studies found that overexpression of miR‐497 decreases cisplatin resistance of ovarian cancer cells in vitro and in vivo 15.

However, whether miR‐497 regulates other target genes to mediate glioma cell growth is still unknown, and the relationship between miR‐497 and chemotherapeutical resistance in glioma cells is not fully elucidated. This study was designed to investigate the molecular mechanisms of miR‐497 in TMZ‐resistant glioma.

## Materials and Methods

### Cell culture and reagents

Six human glioma cell lines (SF295, SHG‐44, U138, LN382, U87, and U251), HEK‐293 T‐cell line, and the normal human astrocytes (NHA) were all purchased from American Type Culture Collection (ATCC, Rockville, MD). The cells were routinely cultured in Dulbecco's Modified Eagle Medium (Gibco, NY) supplemented with 10% fetal bovine serum (Gibco, NY), 0.5% penicillin‐streptomycin, and 1% glutamine at 37°C in a humidified environment containing 5% CO_2_. Antibodies against mammalian target of rapamycin (mTOR), Bcl‐2 (Sigma, St Louis, MO), insulin‐like growth factor 1 receptor *α* (IGF1R*α*), insulin receptor substrate 1 (IRS1) (Abcam, Cambridge, MA), and *β*‐actin (Sigma) were used.

### Generation of stable transformants

Lentiviral constructs containing hsa‐miR‐497 (Lenti‐miR‐497) and anti‐miR‐497 (Lenti‐anti‐miR‐497) along with their matched negative controls (Lenti‐miR‐NC or Lenti‐anti‐miR‐NC) were synthesized by Ambion Austin (TX). Short interfering (siRNA) targeting mTOR and Bcl‐2 were purchased from Thermo Scientific Waltham, MA. as specific oligo pools. mTOR and Bcl‐2 overexpressing vectors and their matched negative controls were purchased from Clontech Laboratories, Inc Mountain View, CA. Briefly, when cells were grown to 40–50% confluence in 6‐well plates, the cells were transfected using lipofectamineTM 2000 (Invitrogen, Carlsbad CA) according to the manufacturer's protocol. Complete media was changed 5 h after transfection.

### Generation of TMZ‐resistant glioma cell lines

To establish the TMZ‐resistant glioma cell lines, U87 and SF295 cells were exposed to gradually increasing concentrations of TMZ (5–100 *μ*mol/L) in culture media for 6 months as described previously 13. The generated TMZ‐resistant cell lines were named as U87‐TR and SF295‐TR.

### Cell viability assay

Cells were seeded in each well of 96‐well plates (5 × 10^3^ cells/well). After overnight incubation, medium was removed and replaced with fresh culture medium. After 48 h of incubation, 10 *μ*L cell counting kit‐8 (CCK‐8) solution (Dojin Laboratory, Kumamoto, Japan) was added to each well 1 h before the end of culture, then the absorbance was measured at 490 nm using a microplate spectrophotometer (Bio‐Tek, Burlington USA).

### Apoptotic cell determination

Cells were harvested and fixed for 20 min in PBS containing 1% glutaraldehyde. Then the cells were permeabilized with 0.1% Triton X‐100 and stained with 1 mmol/L of Hochest 33258, and the nuclei were observed under a fluorescence microscope (Olympus, Tokyo, Japan).

### Quantitative reverse transcription‐polymerase chain reaction (qRT‐PCR)

The total RNA was extracted by Trizol Reagent (Life Technologies, Carlsbad, CA). The cDNA was produced using 1 *μ*g of total RNA according to the protocol for miScript II RT Kit (Qiagen, Hilden, Germany). Real‐time PCR was performed on the 7500 Real‐Time PCR System (Applied Biosystems, Foster City, CA). GAPDH was used as endogenous controls for mRNAs expression. The relative expression of mRNA in each sample was calculated using the 2^∆∆Ct^ method 24.

### Western blot analysis

Cells were harvested with 1× radioimmunoprecipitation assay lysis buffer (Thermo Scientific) with protease inhibitor cocktail (1×; Sigma‐Aldrich St. Louis, Missouri). Homogenates were incubated on ice for 15 min and then centrifuged at 16,099 g for 10 min. The supernatant containing cell proteins was collected and stored at −80°C. Cell lysates were measured by Bradford assay and then were loaded on sodium dodecyl sulfate–polyacrylamide gel electrophoresis gels. Membranes were blocked overnight at 4°C using 5% non‐fat dry milk in Tris‐buffered saline for 1 h and incubated with primary antibodies overnight at 4°C. Afterward, they were probed again with respective secondary antibodies (Sigma). Band signals were visualized using an enhanced chemiluminescence kit (Pierce, Minneapolis, MN). The same membrane was reprobed with the *β*‐actin antibody, which was used as the internal control.

### In vivo tumorigenesis

To confirm the function of miR‐497 in vivo, 1 × 10^7^ logarithmically growing U87 cells stably transfected with lenti‐anti‐NC or lenti‐anti‐miR‐497 were injected subcutaneously into the right armpit of 18–26 g male BALB/c nude mice. Four days after the implantation, the mice were randomly divided into two groups, five mice per group. Mice in both groups were orally treated with 10 mg/kg TMZ for 8 days continuously. At 56 days after inoculation, all mice were killed, while the tumors were excised, weighed, photographed, and subjected to western blot. All animal procedures were approved by the Ethics Animal Care and Use Committee of The First Affiliated Hospital of Wenzhou Medical University.

### Immunolocalization of Ki‐67 in tumor samples

Xenograft tumor tissues of all mice from both the anti‐NC and anti‐miR‐497 groups were embedded in paraffin and fixed with formaldehyde. Such samples were sectioned, and immunostaining was performed using specific antibody against Ki‐67 with appropriate dilution and using normal host serum for negative control, followed by staining with appropriate HRP‐conjugated secondary antibody. The slides were developed in 3‐3′‐diaminobenzidinetetrahydrochloride solution and counterstained with a weak hematoxylin solution stain. The stained sections were counted in 10 random views at 400× magnification by two different pathologists who remained blind to the xenograft tumor groups. Staining intensity was semiquantified in 20 randomly selected fields per tumor, and the proliferation index was expressed as Ki‐67‐positive cells/total cells in the view × 100%.

### TUNEL labeling assay

Apoptotic cells were detected using the in situ cell death assay kit from Roche diagnostics as per manufacturer's instructions. The apoptosis index was calculated as the percentage of terminal deoxyribonucleotidyl transferase‐mediated dUTP nick end labeling (TUNEL)‐labeled cells and was obtained by counting 20 randomly chosen visual fields of the most affected tumor areas under a microscope.

### Statistical analysis

Data were expressed as mean ± standard deviation values or percent of controls. Statistical differences were evaluated by one‐way analysis of variance (ANOVA) followed by dunnett‐t multiple comparisons tests. All calculations were performed with the SPSS software (version 15.0, SPSS, Inc. Chicago, Illinois). *P* < 0.05 was considered to be statistically significant.

## Results

### Establishment and characterization of TMZ‐resistant glioma cell lines

**Figure 1:**
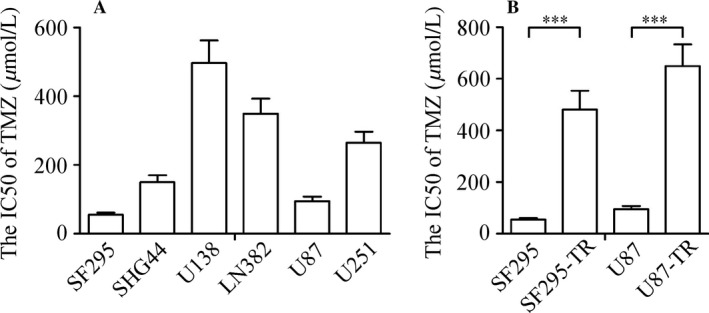
The establishment of TMZ‐resistant glioma cell lines. (A) The IC50 values of TMZ in six glioma cell lines. (B) The IC50 values of TMZ were markedly higher in TMZ‐resistant glioma cell lines than their parental cell lines. Results are reported as the mean ± SD of four independent experiments, ***P <* *0.001. TMZ, temozolomide; SD, standard deviation.

To elucidate the TMZ resistance in glioma cell lines, the initial experiment was focused on the sensitivity of TMZ in six glioma cell lines. Our results showed that two of them SF295 and U87, exhibited a lower IC50 than the other cell lines U138, LN382, SHG‐44, and U251 (Fig. 1A). Next, by exposing U87 and SF295 cells to increasing concentrations of TMZ for 6 months, we were able to establish two resistant glioma cell lines to TMZ (U87‐TR and SF295‐TR). Thus, this resistance phenotype was stable and cell autonomous, as it was not reversed by culturing U87‐TR or SF295‐TR cells in TMZ‐free medium for up to 6 months. These U87‐TR and SF295‐TR cell lines are ~9 times and 7 times resistant to TMZ compared with their parental cell lines, respectively (Fig. 1B).

### Analysis of miR‐497 in glioma cell lines

**Figure 2:**
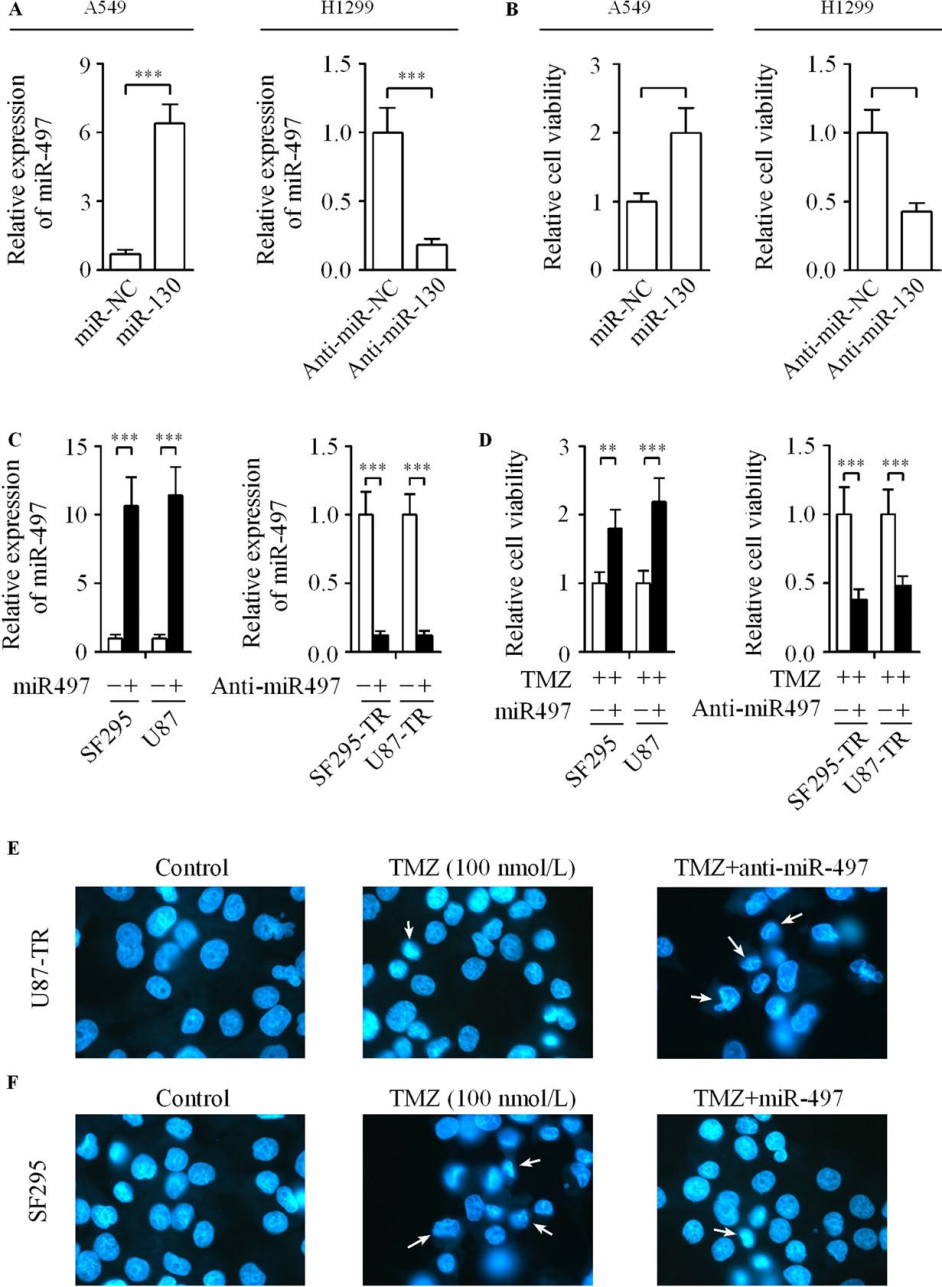
Effect of miR‐497 on the resistance of glioma cells to TMZ treatment. (A) Relative level of miR‐497 was significantly increased in six human glioma cell lines than in NHA using qRT‐PCR. (B) The level of miR‐497 was detected in SF295‐TR and U87‐TR cells as well as their matched parental cell lines. (C) Relative expression of miR‐497 in glioma cells transfected with the miR‐497, anti‐miR‐497 or their matched controls. (D) CCK‐8 assay was used to evaluate the effect of miR‐497 on the cell growth of glioma cells. (E) U87‐TR cells were induced by TMZ (100 nmol/L) in the presence or absence of anti‐miR‐497, the down‐regulation of miR‐497 promoted apoptosis induced by TMZ in glioma cells. (F) The apoptosis in SF295 cells induced by TMZ was inhibited by the up‐regulation of miR‐497, the arrows show the typical apoptotic cells. Data were derived from three experiments with six replicates. **P <* *0.01, ***P <* *0.001. TMZ, temozolomide; NHA, normal human astrocytes; qRT‐PCR, quantitative reverse transcription‐polymerase chain reaction; CCK‐8, cell counting kit‐8.

To investigate the function of miR‐497 on TMZ sensitivity in glioma cells, we examined the relative expression of miR‐497 in several glioma cell lines as well as NHA, which is a NHA by qRT‐PCR. The results demonstrated that the expression of miR‐497 was increased in all the six glioma cell lines than that in NHA (Fig. 2A). Furthermore, the correlation between the relative miR‐497 level and IC50 values was analyzed. Interestingly, the abundance of miR‐497 was positively correlated with IC50 values in glioma cell lines. Investigations into such information indicated that miR‐497 was closely associated with TMZ sensitivity in glioma cells. We next evaluated whether acquired resistance to TMZ impacted the level of miR‐497 in glioma cells. Our results show that the level of miR‐497 was up‐regulated in U87‐TR and SF295‐TR cell lines compared with their matched parental cell lines (Fig. 2B).

### Effect of miR‐497 on the TMZ resistance of glioma cells

To determine the role of miR‐497 in TMZ‐resistant phenotype of glioma cells, the expression of miR‐497 in U87‐TR and SF295‐TR cells with high endogenous miR‐497 levels was down‐regulated by the transfection of cells with anti‐miR‐497 or anti‐miR‐NC. We also promoted the expression of miR‐497 in U87 and SF295 cells after transfecting with miR‐497 or negative control. As a result, the expression of miR‐497 was decreased significantly after the glioma cells were transfected with anti‐miR‐497. Moreover, transfection of miR‐497 led to a significant increase in its expression (Fig. 2C). Additionally, the effects of miR‐497 or anti‐miR‐497 on the proliferation of glioma cells were investigated. As shown in Figure 2D, the result of CCK‐8 assay indicated that the down‐regulation of miR‐497 significantly reduced U87‐TR and SF295‐TR cells resistance to TMZ. On the contrary, the up‐regulation of miR‐497 dramatically increased U87 and SF295 cells tolerance to TMZ. We further examined the cellular apoptosis by Hochest 33258 staining assay in miR‐497‐depleted U87‐TR and miR‐497‐overexpressed SF295 cells. Our data showed clearly that the inhibition of miR‐497 promoted apoptosis induced by TMZ in U87‐TR cells (Fig. 2E). However, the ectopic miR‐497 prevented the percentage of apoptotic cells in SF295 cells induced by TMZ (Fig. 2F). These data suggest that miR‐497 plays a crucial role in the development of TMZ resistance in glioma cells in vitro.

### MiR‐497 directly targets mTOR/Bcl‐2

**Figure 3:**
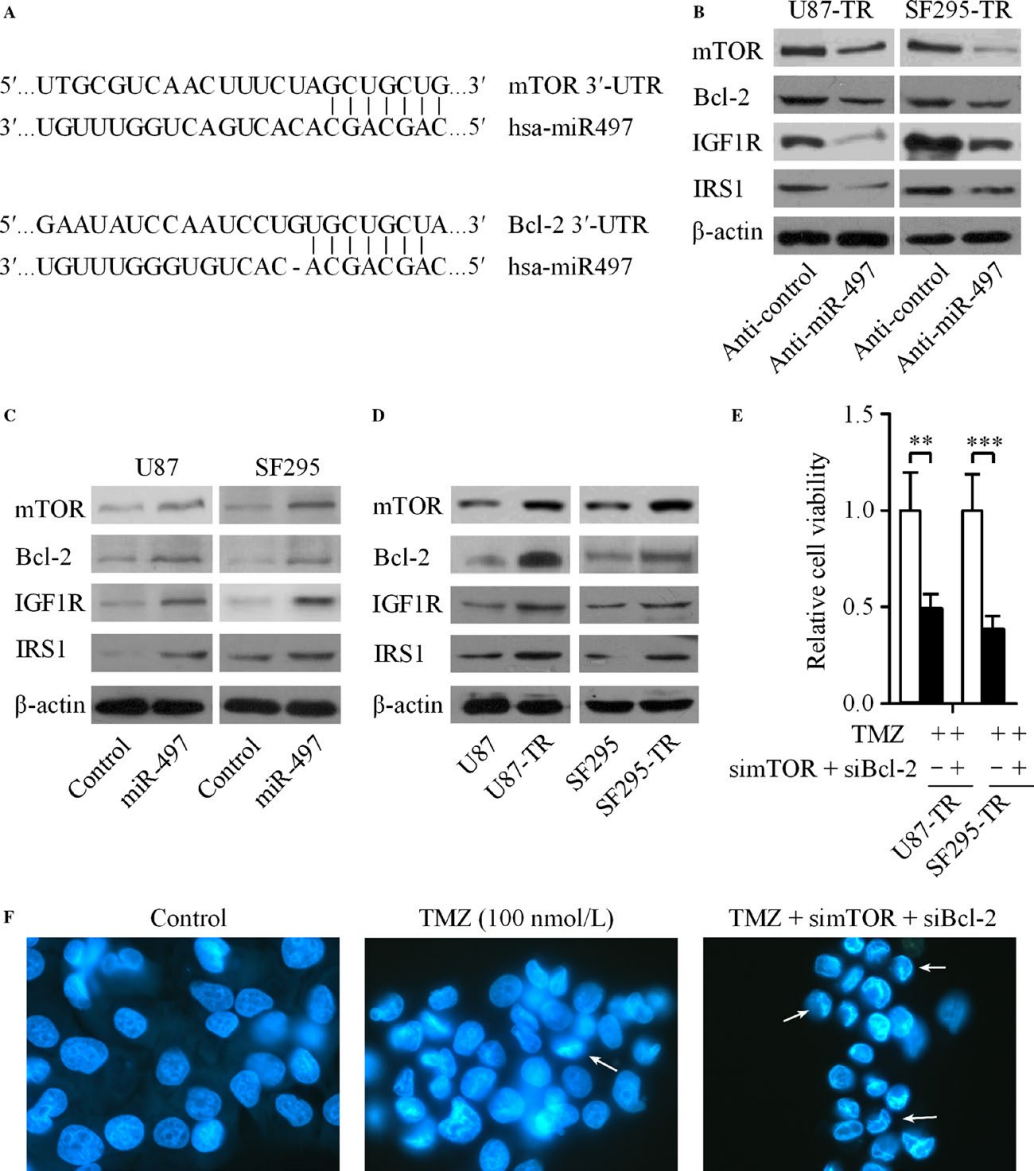
MiR‐497 directly targets mTOR and Bcl‐2. (A) The putative miR‐497 binding sequences in the 3′‐UTR of mTOR and Bcl‐2. (B) The levels of IGF1R, IRS1, mTOR, and Bcl‐2 in U87‐TR and SF295‐TR cells after transfecing with anti‐miR‐497 were analyzed by western blot. (C) The expression of IGF1R, IRS1, mTOR, and Bcl‐2 in U87 and SF295 cells was up‐regulated by the transfection of miR‐497. (D) Relative expression levels of IGF1R/IRS1 pathway‐related proteins were increased in U87‐TR and SF295‐TR cells than their matched parental cell lines. (E) Knockdown of endogenous mTOR and Bcl‐2 in U87‐TR and SF295‐TR cells markedly decreased TMZ‐resistance of glioma cells. (F) Silencing of mTOR and Bcl‐2 promoted the apoptosis of U87‐TR cells induced by TMZ, the arrows shown the typical apoptotic cells. Results are reported as the mean ± SD of three independent experiments, **P <* *0.01, ***P <* *0.001. mTOR, mammalian target of rapamycin; 3′‐UTR, 3′‐untranslated region; IGF1R, insulin‐like growth factor 1 receptor; IRS1, insulin receptor substrate 1; TMZ, temozolomide; SD, standard deviation.

To explore the molecular mechanisms of miR‐497 in regulation of cell growth and apoptosis in glioma cells. Three well‐developed bioinformatics algorithms (RNAhybrid 2.1, TargetScan, and PicTar) predicted that miR‐497 targets mTOR and Bcl‐2 (Fig. 3A), which had been reported as targets in ovarian and breast cancer cells 12, 18. To confirm whether endogenous mTOR and Bcl‐2 are regulated by miR‐497, the protein expression levels of mTOR and Bcl‐2 were assessed in glioma cells transfected with miR‐497, anti‐miR‐497 and their matched controls. The silencing of miR‐497 decreased the protein levels of IGF1R/IRS1 pathway‐related proteins, that is, IGF1R, IRS1, mTOR, and Bcl‐2 (Fig. 3B). However, the levels of the above IGF1R/IRS1 signal pathway‐related proteins were increased when U87 and SF295 cells were transfected with miR‐497 (Fig. 3C).

### MiR‐497 increases TMZ resistance by targeting mTOR/Bcl‐2

**Figure 4:**
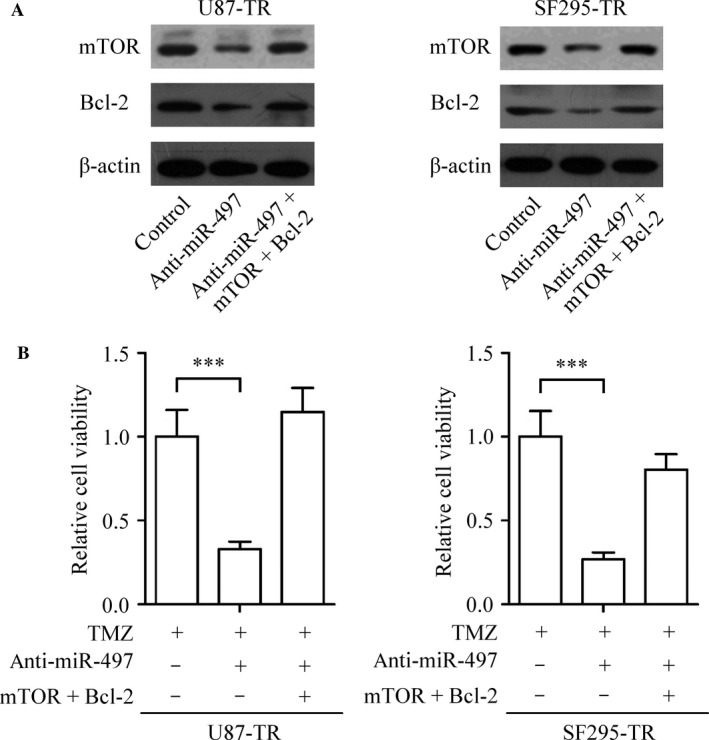
MiR‐497 inhibits TMZ resistance by targeting mTOR and Bcl‐2 in glioma cells. (A) The levels of mTOR and Bcl‐2 in U87‐TR and SF295‐TR cells after transfecting with anti‐miR‐497 with or without mTOR and Bcl‐2 overexpressing vectors were analyzed by western blot. (B) U87‐TR and SF295‐TR cells were maintained in the medium with or without anti‐miR‐497 and were infected using mTOR and Bcl‐2 overexpressing vectors. Then the cells were treated with TMZ before CCK‐8 assay. Results are reported as the mean ± SD of three independent experiments, ***P <* *0.001. TMZ, temozolomide; mTOR, mammalian target of rapamycin; CCK‐8, cell counting kit‐8; SD, standard deviation.

Our result revealed that the protein levels of IGF1R, IRS1, mTOR, and Bcl‐2 were increased in TMZ‐resistant glioma cells (U87‐TR and SF295‐TR) compared with TMZ‐sensitive glioma cells (U87 and SF295) (Fig. 3D). Since mTOR and Bcl‐2 were associated with the IGF1R/IRS1 signal pathway, along with the confirmation of mTOR and Bcl‐2 are direct targets of miR‐497. We hypothesized that miR‐497 could regulate TMZ resistance in glioma cells by targeting IGF1R/IRS1 pathway. To verify this, we performed mTOR and Bcl‐2 loss‐of function experiments in U87‐TR and SF295‐TR cells to investigate whether the silencing of mTOR and Bcl‐2 in glioma cells could regulate the chemotherapy resistance of glioma cells in vitro. The knockdown of mTOR and Bcl‐2 exerted a similar effect as depletion of miR‐497 on reducing both glioma cell lines resistance to TMZ treatment (Fig. 3E). In addition, we further investigated the cellular apoptosis in mTOR and Bcl‐2 knockdown U87‐TR cells. Our results showed that the loss of mTOR and Bcl‐2 increased the apoptotic cells induced by TMZ in U87‐TR cells (Fig. 3F). Next, we successfully acquired mTOR and Bcl‐2‐overexpressed glioma cells. Interestingly, enhanced levels of mTOR and Bcl‐2 obviously abrogated the anti‐resistant effect induced by anti‐miR‐497 (Fig. 4). These data showed that the up‐regulation of miR‐497 promotes acquisition of TMZ‐resistant ability in glioma cell via promotion of IGF1R/IRS1 signal pathway‐related proteins.

### MiR‐497 increases TMZ resistance of glioma cells in vivo

**Figure 5:**
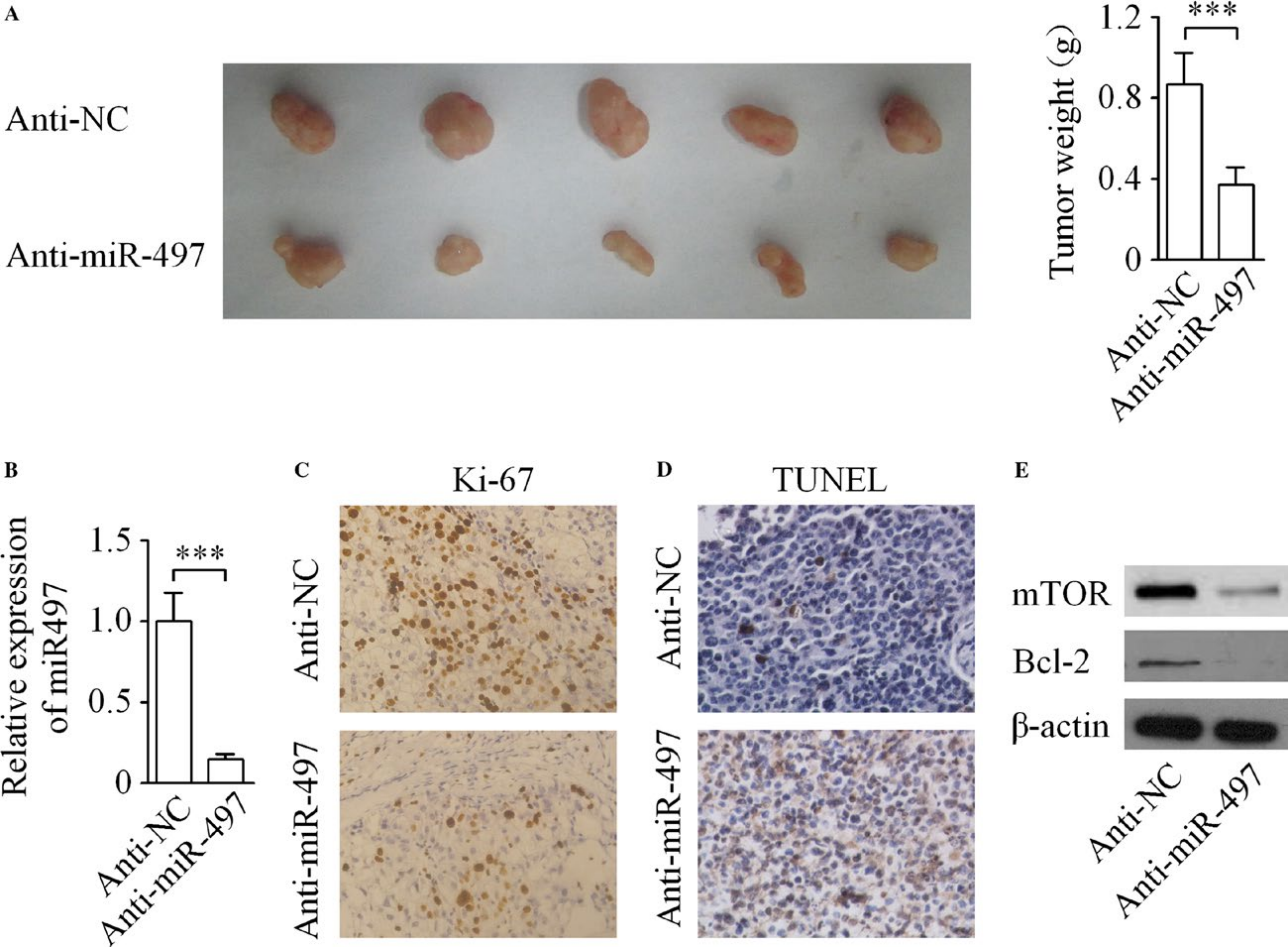
Down‐regulation of miR‐497 significantly suppressed the growth of subcutaneous tumor. (A) Necropsy photographs of tumor‐bearing mice showing therapeutic benefit of miR‐497 depletion. (B) The relative level of miR‐497 was analyzed in tumor tissues by qRT‐RCR. (C) Down‐regulation of miR‐497 remarkably reduced Ki‐67‐positive expression in xenograft tumors established by U87‐TR cells. (D) Apoptosis of glioma cells in vivo was detected by TUNEL assay. (E) Western blot showing that the expression of mTOR and Bcl‐2 were decreased in U87‐TR/lenti‐anti‐miR‐497 cells formed tumors. ***P <* *0.001. TUNEL, terminal deoxyribonucleotidyl transferase‐mediated dUTP nick end labeling; mTOR: mammalian target of rapamycin.

To indentify whether down‐regulation of miR‐497 in TMZ‐resistant glioma cells could enhance the sensitivity of TMZ in *vivo*”. At the end of experiment, the subcutaneous xenograft tumors were excised and weighed. As shown in Figure 5A, consistent with the results obtained from the in vitro assays, the xenograft tumor weights were indicative of a significant decrease in the U87‐TR/lenti‐anti‐miR‐497 + TMZ (anti‐miR‐497 + TMZ) group in comparison with U87‐TR/lenti‐anti‐NC + TMZ (anti‐NC + TMZ) group. The level of miR‐497 was down‐regulated in anti‐miR‐497 + TMZ group tumor tissues than that in anti‐NC + TMZ group tumor tissues (Fig. 5B).

### Immunohistochemical staining in vivo

Then, the immunohistochemical detection of cell proliferation marker Ki‐67 expression in the tumor tissues was performed. As indicated in Figure 5C, the percentage of Ki‐67‐positive stained cells was significantly higher in the tumor derived from anti‐NC + TMZ group than its expression in the anti‐miR‐497 + TMZ group. In addition, quantitative data showed that TUNEL staining of these tissue sections indicated significant differences in the percentage of TUNEL‐positive cells in the tumors derived from the anti‐miR‐497 + TMZ group compared with the anti‐NC + TMZ group (Fig. 5D). Subsequently, the levels of mTOR and Bcl‐2 in the xenograft tumors of both groups were examined by western blot. Our results demonstrated that the expression of mTOR and Bcl‐2 were markedly decreased in U87‐TR/lenti‐anti‐miR‐497 cells formed tumors compared with that in U87‐TR/lenti‐anti‐NC cells formed tumors at the translational level (Fig. 5E).

## Discussion

Previous studies have demonstrated that miRNAs are up‐regulated or down‐regulated in glioma samples, and associated with histological subtypes, tumor stages, recurrent tumors, and overall survival 7, 12, 13, 19, 20. Recently, increasing evidence also showed that miRNAs are involved in the regulation of glioma cells chemotherapy or radiotherapy resistance 21, 22. However, to the best of our knowledge, there is limited information regarding the contribution that miRNAs make to the chemoresistance of glioma and the mechanisms of miRNAs in regulating resistance of TMZ in glioma remains poorly understood. In this study, qRT‐PCR analysis showed that the expression of miR‐497 is significantly up‐regulated in several glioma cell lines in comparison with normal cells. The differential expression of miR‐497 was indicative of the fact that miR‐497 might be involved in the carcinogenesis of glioma. Our results are consistent with a recent study which demonstrated that miR‐497 expression is associated with cervical cancer growth and as a novel noninvasive biomarker for detection of cervical cancer 16.

In this study, the level of miR‐497 in glioma cells was interfered with an effort to explore the function of miR‐497 on the TMZ resistance of glioma cells. Our results demonstrated that the level of miR‐497 was markedly up‐regulated in both TMZ‐resistant glioma cell lines than their matched parental cell lines. Furthermore, CCK‐8 assay and apoptosis assay supported our hypothesis that the down‐regulation of miR‐497 can enhance the cytotoxic effect of TMZ on glioma cells, and vice versa. Thus, our data were collectively suggestive of the idea that miR‐497 plays a vital role in TMZ resistance of glioma cells in vitro. Further trials were employed to validate the hypothesis that the impact of miR‐497 on the TMZ resistance of glioma is sustained in vivo. In vivo results showed that the down‐regulation of miR‐497 in U87‐TR cells significantly increases TUNEL staining and decreases Ki‐67 immunoreactivity, which is indicative of the apoptosis and reduced cellular proliferation within tumors, leading to a much better TMZ treatment effect on glioma xenograft tumor growth than control group. These in vitro and in vivo findings provides promising evidence supporting miR‐497 as a valuable future target for glioma treatment, and miR‐497 might be used as a “supplement” in TMZ‐based chemotherapy. Applied TMZ with miR‐497 antagonists may help to reduce the rate of TMZ‐resistant glioma occurrence and improving the overall response rate to chemotherapy.

Numerous studies have demonstrated that the IGF1R/IRS1 pathway was involved in cell proliferation and metastasis control of several solid tumors 23, 24. mTOR is associated with various biological processes including cell proliferation, differentiation, migration and invasion, angiogenesis, survival as well as tumorigenesis 25, 26. Recent studies demonstrated that mTOR also plays an important role in chemotherapeutic resistance 27, 28. Moreover, PI3K/Akt/mTOR signaling pathway is associated with prostate cancer radioresistance 29. The Bcl‐2 family proteins play a crucial role in apoptosis through the balance of pro‐apoptotic proteins and anti‐apoptotic proteins 30. Bcl‐2 has been shown to modulate chemosensitivity in several human cancers 31, 32. Recent trials demonstrated that several miRs mediate chemotherapy resistance by targeting mTOR or Bcl‐2 12, 13, 15, 18, 30. However, the association of miR‐497 and chemosensitivity of IGF1R/IRS1 pathway has not yet been explored in glioma. Our results demonstrated that the down‐regulation of miR‐497 could inhibit the levels of IGF1R/IRS1 signal pathway‐related proteins in TMZ‐resistant glioma cells both in vitro and in vivo. Furthermore, overexpression of miR‐497 markedly increased the expression of IGF1R/IRS1 signal pathway‐related proteins in TMZ‐sensitive cells, which hence became resistant to TMZ‐based chemotherapy. Moreover, loss‐of‐function analyses disclosed that inhibition of mTOR and Bcl‐2 suppresses the TMZ resistance of TMZ‐resistant cells, consistent with the effects of miR‐497 depletion in the same cells. In addition, our current finding showed that mTOR and Bcl‐2 were regulated by miR‐497 directly, highlighting the potential role of miR‐497 overexpression for glioma to develop a TMZ‐resistance phenotype. We demonstrated that miR‐497 could regulate TMZ resistance in glioma cells by promoting cell proliferation and inhibiting apoptosis, at least partially, via targeting the IGF1R/IRS1 pathway.

## Conclusion

In conclusion, our results are consistent with the hypothesis that miR‐497 as a glioma promoter, is significantly correlated with the TMZ resistance of human glioma cells by targeting the IGF1R/IRS1 pathway both in vitro and in vivo. Moreover, mTOR and Bcl‐2 were identified as direct physiological targets of miR‐497 with biological function that are implicated in the development of TMZ‐resistance phenotype. This work highlights the therapeutic potential of miR‐497 in the functional regulation of glioma.

## Conflict of Interest

None declared.
